# Bisphenol A Induces Gene Expression Changes and Proliferative Effects through GPER in Breast Cancer Cells and Cancer-Associated Fibroblasts

**DOI:** 10.1289/ehp.1104526

**Published:** 2012-05-02

**Authors:** Marco Pupo, Assunta Pisano, Rosamaria Lappano, Maria Francesca Santolla, Ernestina Marianna De Francesco, Sergio Abonante, Camillo Rosano, Marcello Maggiolini

**Affiliations:** 1 Department of Pharmaco-Biology, University of Calabria, Rende, Italy; 2 Regional Hospital, Cosenza, Italy; 3 Department of Bioinformatics and Structural Proteomics, National Institute for Cancer Research, Genova, Italy

**Keywords:** bisphenol A, breast cancer cells, cancer-associated fibroblasts, GPR30/GPER, tumor microenvironment

## Abstract

Background: Bisphenol A (BPA) is the principal constituent of baby bottles, reusable water bottles, metal cans, and plastic food containers. BPA exerts estrogen-like activity by interacting with the classical estrogen receptors (ERα and ERβ) and through the G protein-coupled receptor (GPR30/GPER). In this regard, recent studies have shown that GPER was involved in the proliferative effects induced by BPA in both normal and tumor cells.

Objectives: We studied the transduction signaling pathways through which BPA influences cell proliferation and migration in human breast cancer cells and cancer-associated fibroblasts (CAFs).

Methods and results: We used as a model system SKBR3 breast cancer cells and CAFs that lack the classical ERs. Specific pharmacological inhibitors and gene-silencing procedures were used to show that BPA induces the expression of the GPER target genes c-*FOS*, *EGR-1*, and *CTGF* through the GPER/EGFR/ERK transduction pathway in SKBR3 breast cancer cells and CAFs. Moreover, we observed that GPER is required for growth effects and migration stimulated by BPA in both cell types.

Conclusions: Results indicate that GPER is involved in the biological action elicited by BPA in breast cancer cells and CAFs. Hence, GPER-mediated signaling should be included among the transduction mechanisms through which BPA may stimulate cancer progression.

Bisphenol A (BPA), used largely in the manufacture of polycarbonate plastics, is the constituent of a wide array of consumer products, including plastic food containers, baby bottles, and the lining of metal food cans (Welshons et al. 2006). Humans are exposed to BPA mainly at the time of consumption of water and foods through the materials used for containers and packages (Vandenberg et al. 2009).

BPA is able to accelerate growth and puberty, alter the ovarian cycle in females (Mlynarcíková et al. 2005; Rasier et al. 2006), interfere with embryonic development, and to induce aneuploidy (Takai et al. 2000). Moreover, a relationship between BPA blood levels, obesity, polycystic ovary syndrome, repeated miscarriage, and endometrial hyperplasia has been found in women, suggesting that it may act as an endocrine disruptor (Welshons et al. 2006). Exposure to BPA has also been correlated with the incidence of diverse types of tumors (Ho et al. 2006; Keri et al. 2007; Maffini et al. 2006).

BPA has estrogenic activity both *in vivo* and *in vitro* and is thought to be an environmental estrogen (Welshons et al. 2006). Previous investigations (reviewed by Vandenberg et al. 2009) have demonstrated that BPA binds to and activates the estrogen receptor (ERα and ERβ), although the affinity of BPA for these receptors was approximately 10,000-fold weaker with respect to estradiol (Gould et al. 1998; Kuiper et al. 1998). In recent years, the identification of G protein-coupled receptor (GPER) as a novel estrogen receptor has suggested new possibilities by which estrogenic compounds might cause biological effects in different cell types (Albanito et al. 2007; Maggiolini et al. 2004; Prossnitz and Maggiolini 2009; Vivacqua et al. 2006a, 2006b). In this regard, we reported a characteristic signature elicited by estrogenic GPER signaling in SKBR3 breast cancer cells and we identified a network of transcription factors, such as *c-FOS*, early growth response protein 1 (*EGR-1*), and connective tissue growth factor (*CTGF*), that may be involved in important biological functions (Pandey et al. 2009).

BPA is one of several environmental estrogens that have exhibited the ability to bind to GPER (Thomas and Dong 2006) and to activate transduction pathways (Dong et al. 2011) involved in the biological responses of both normal and neoplastic cells. For example, BPA stimulated the proliferation of mouse spermatogonial cells (Sheng and Zhu 2011) and human seminoma cells (Bouskine et al. 2009) and induced chemoresistance in breast cancer cells (Lapensee et al. 2009) through activation of GPER.

The contribution of the stromal microenvironment to the development of a wide variety of tumors has been highlighted by clinical evidence and the use of mouse models (Bhowmick et al. 2004a). A growing body of data has also suggested that tumor cells actively recruit cancer-associated fibroblasts (CAFs), which remain activated and play a prominent role in cancer progression (Bhowmick et al. 2004b). In breast carcinoma approximately 80% of stromal fibroblasts may acquire the activated phenotype that promotes the proliferation of cancer cells at metastatic sites, stimulating tumor growth such as for the primary tumor (Kalluri and Zeisberg 2006).

In this study, we demonstrate that BPA exerts a stimulatory action through GPER in breast cancer cells and CAFs.

## Materials and Methods

*Reagents.* We purchased bisphenol A (BPA), *N*-[2-(*p*-bromocinnamylamino)ethyl]-5-soquinolinesulfonamide dihydrochloride (H89), PD98059 (PD), and arsenic trioxide (As_2_O_3_) from Sigma-Aldrich (Milan, Italy); AG1478 (AG) from Biomol Research Laboratories (DBA, Milan, Italy), and 1-(4-(6-bromobenzo[1,3]dioxol-5-yl)-3a,4,5,9b-tetrahydro-3H-cyclopenta[*c*]quinolin-8-yl)-ethanone (G-1) from Calbiochem (Merck KGaA, Frankfurt, Germany). As_2_O_3_ was dissolved in phosphate-buffered saline, and BPA and PD were dissolved in ethanol; AG1478, H89, and G-1 were solubilized in dimethyl sulfoxide (DMSO).

*Cell culture.* SKBR3 cells. SKBR3 human breast cancer cells were maintained in phenol red-free RPMI 1640 medium supplemented with 10% fetal bovine serum (FBS). Cells were changed to medium without serum the day before experiments for immunoblotting and reverse-transcription polymerase chain reaction (RT-PCR).

CAFs. CAFs were extracted as previously described (Madeo and Maggiolini 2010). Briefly, breast cancer specimens were collected from primary tumors of patients who had undergone surgery. Signed informed consent was obtained from all the patients and from the institutional review board(s) of the Regional Hospital of Cosenza. Tissues from tumors were cut into smaller pieces (1–2 mm diameter), placed in digestion solution (400 IU collagenase, 100 IU hyaluronidase, and 10% serum, containing antibiotic and antimycotic solution), and incubated overnight at 37°C. The cells were then separated by differential centrifugation at 90 × *g* for 2 min. Supernatant containing fibroblasts was centrifuged at 485 × *g* for 8 min; the pellet obtained was suspended in fibroblasts growth medium (Medium 199 and Ham’s F12 mixed 1:1 and supplemented with 10% FBS) and cultured at 37°C in 5% CO_2_. Primary cells cultures of breast fibroblasts were characterized by immunofluorescence. Briefly cells were incubated with human anti-vimentin (V9) and human anti-cytokeratin 14 (LL001), both from Santa Cruz Biotechnology DBA (Milan, Italy). To assess fibroblasts activation, we used anti-fibroblast activated protein α (FAPα) antibody (H-56; Santa Cruz Biotechnology DBA) (data not shown).

*Western blotting.* SKBR3 cells and CAFs were grown in 10-cm dishes, exposed to treatments or ethanol (or DMSO), which was used as the vehicle, and then lysed as described previously (Pandey et al. 2009). Protein concentrations were determined using Bradford reagent (Sigma-Aldrich) according to the manufacturer’s recommendations. Equal amounts of whole protein extract were resolved on a 10% SDS-polyacrylamide gel and transferred to a nitrocellulose membrane (Amersham Biosciences, Milan, Italy). Membranes were probed overnight at 4°C with antibodies against c-Fos (H-125), β-actin (C-2), phosphorylated extracellular signal-regulated kinase 1/2 (p-ERK1/2; E-4), Egr-1 (588), CTGF (L-20), ERK2 (C-14), ERα (F-10), or GPR30 (N-15), all from Santa Cruz Biotechnology, DBA (Milan, Italy), or ERβ from Serotec (Space Import Export, Milan, Italy). Results of densitometric analyses of Western blots, obtained using ImageJ software (Abramoff et al. 2004), are presented as optical density (OD; expressed in arbitrary units) relative to the control (ERK2 or β-actin).

*Plasmids and luciferase assays.* The *Ctgf* luciferase reporter plasmid p(-1999/+36)-luc, which is based on the backbone of vector pGL3-basic (Promega), was a gift from B. Chaqour (Department of Anatomy and Cell Biology, State University of New York Downstate Medical Center, Brooklyn, NY, USA). The luciferase reporter plasmid for *c-FOS* encoding a –2.2-kb 5´ upstream fragment of human *c-FOS* was a gift from K. Nose (Department of Microbiology, Showa University School of Pharmaceutical Sciences, Hatanodai, Shinagawa-ku, Tokyo, Japan). The *EGR-1* luciferase reporter plasmid pEgr-1A, which contains the –600 to +12 5´-flanking sequence from the human *EGR-1* gene was a gift from S. Safe (Department of Veterinary Physiology and Pharmacology, Texas A&M University, Houston, TX, USA). For the luciferase assays, cells were transferred into 24-well plates containing 500 μL of regular growth medium per well the day before transfection. On the day of transfection, SKBR3 cell medium was replaced with RPMI without phenol red and serum, and transfection was performed using Fugene6 Reagent (Roche Molecular Biochemicals, Milan, Italy) and a mixture containing 0.5 μg of each reporter plasmid. Renilla luciferase (pRL-CMV; 1 ng) was used as a transfection control. After 5–6 hr, ligand was added and cells were incubated for 16–18 hr. We measured luciferase activity using the Dual Luciferase Kit (Promega, Milan, Italy) according to the manufacturer’s recommendations. Firefly luciferase values generated by the reporter plasmid were normalized to Renilla luciferase values. Normalized values obtained from cells treated with ethanol vehicle were set as 1-fold induction, and the activity induced by treatments was calculated based on this value.

*RT-PCR and real-time PCR.* Total RNA was extracted using Trizol commercial kit (Invitrogen, Milan, Italy) according to the manufacturer’s protocol. RNA was quantified spectrophotometrically, and cDNA was synthesized from the RNA by reverse transcription using murine leukemia virus reverse transcriptase (Invitrogen). We quantified the expression of selected genes by real-time PCR using SYBR Green as the detection method and the Step One sequence detection system (Applied Biosystems Inc., Milan, Italy). Gene-specific primers were designed using Primer Express software (version 2.0; Applied Biosystems Inc.). Assays were performed in triplicate. We used mean values to calculate expression levels by the relative standard curve method. For the sequences of primer used, see Supplemental Material, Table S1(http://dx.doi.org/10.1289/ehp.1104526).

*Gene silencing experiments.* Cells were plated onto 10-cm dishes, maintained in serum-free medium for 24 hr, and then transfected for an additional 24 hr before treatments using Fugene6. The short hairpin (sh) RNA constructs to knock down the expression of *GPER* and *CTGF* and the unrelated shRNA control construct have been described previously (Pandey et al. 2009).

*Wound-healing assay.* CAFs were seeded into 12-well plates in regular growth medium. When at 70% to 80% confluency, the cells were transfected with shGPER using Fugene6 reagent for 24 hr. Transfected cells were washed once, medium was replaced with 2.5% charcoal-stripped FBS, and cells were treated. We then used a p200 pipette tip to scratch the cell monolayer. In experiments performed using conditioned medium, CAFs were plated into 12-well plates and transfected with 500 ng shRNA control plasmid or shGPER or shCTGF plasmids using Fugene6, as recommended by the manufacturer. After 24 hr, CAFs were treated with 1 μM BPA, and the conditioned medium was collected and filtered through a sterile nonpyrogenic 0.2 μm filter. The conditioned medium obtained was added to subconfluent SKBR3 cells, and a series of scratches were made using a p200 pipette tip. We evaluated cell migration in three independent experiments after 48 hr of treatment; data are expressed as a percentage of cells in the wound area upon treatment compared with cells receiving vehicle.

*Proliferation assay.* SKBR3 cells and CAFs were seeded in 24-well plates in regular growth medium. After cells attached, they were washed, incubated in medium containing 2.5% charcoal-stripped FBS, and transfected with 500 ng shGPER or control shRNA plasmids using Fugene6 reagent. After 24 hr, cells were treated with 1 μM BPA, and the transfection and treatment were renewed every 2 days. We counted the cells using the COUNTESS automated cell counter (Invitrogen) following the manufacturer’s recommendations.

*Statistical analysis.* For statistical analysis, we used analysis of variance followed by Newman-Keuls testing to determine differences in means. *p*-Values < 0.05 are considered statistically significant.

## Results

**Figure 1:**
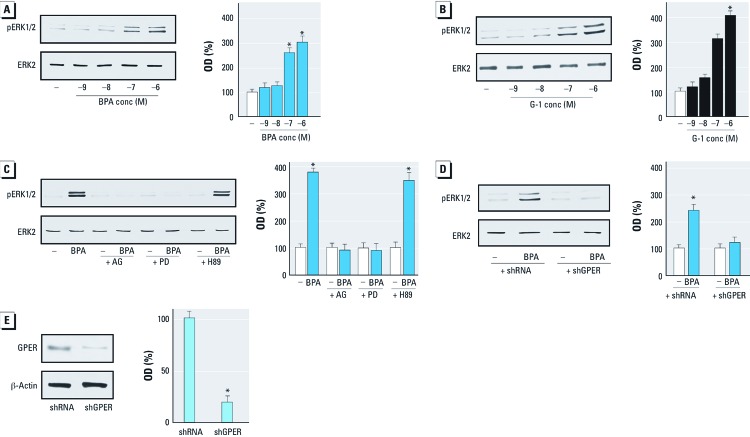
Induction of ERK1/2 phosphorylation (pERK1/2) by BPA and G‑1 through GPER in SKBR3 cells. conc, concentration. (*A*,*B*) Cells were treated for 30 min with vehicle (–) or increasing concentrations of BPA (*A*) or G‑1 (*B*). (*C*) ERK1/2 phosphorylation in SKBR3 cells treated for 30 min with vehicle or 1 μM BPA alone or in combination with 10 µM AG1478, PD, or H89 (inhibitors of EGFR, MEK, and PKA, respectively). (*D*) ERK1/2 phosphorylation in SKBR3 cells transfected with shRNA or shGPER and treated with vehicle or 1 μM BPA for 30 min. (*E*) Efficacy of GPER silencing. Graphs show densitometric analyses of the blots normalized to ERK2 (*A–D*) or β-actin (*E*); values shown represent the mean ± SD of three independent experiments. *p < 0.05 compared with vehicle.

**Figure 2:**
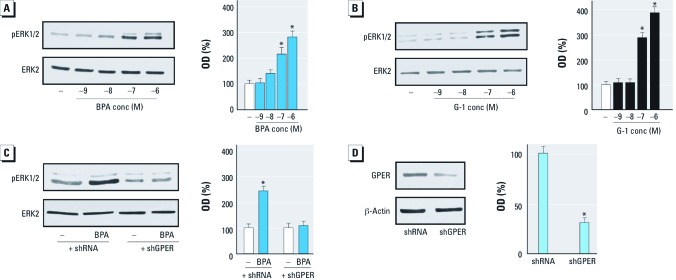
Induction of ERK1/2 phosphorylation (pERK1/2) by BPA and G‑1 through GPER in CAFs. conc, concentration. (*A*,*B*) CAFs were treated for 30 min with vehicle (–) or increasing concentrations of BPA (*A*) or G‑1 (*B*). (*C*) ERK1/2 phosphorylation in CAFs transfected with shRNA or shGPER and treated with vehicle or 1 μM BPA for 30 min. (*D*) Efficacy of GPER silencing in CAFs. Graphs show densitometric analyses of the blots normalized to ERK2 (*A–C*) or β-actin (*D*); values shown represent the mean ± SD of three independent experiments. *p < 0.05 compared with vehicle.

*BPA induces ERK1/2 activation through GPER.* Using SKBR3 cells and CAFs, which both express GPER and lack ERs [see Supplemental Material, Figure S1 (http://dx.doi.org/10.1289/ehp.1104526)], we evaluated ERK1/2 activation by increasing concentrations of BPA and the GPER ligand G-1, as GPER activation leads to ERK1/2 phosphorylation (Dong et al. 2011; Maggiolini and Picard 2010). BPA and G-1 induced ERK1/2 phosphorylation in both cell types in a dose-dependent manner (Figures 1A,B and 2A,B). When the epidermal growth factor receptor (EGFR) inhibitor AG1478 or the mitogen-activated protein kinase kinase MEK inhibitor PD was added, ERK1/2 activation was not evident, but it was present when the protein kinase A (PKA) inhibitor H89 was added (Figure 1C). Interestingly, ERK1/2 phosphorylation by BPA was abolished by silencing GPER expression (Figures 1D, 2C), suggesting that GPER is required for ERK1/2 activation after exposure to BPA. We ascertained the efficacy of GPER silencing using immunoblots in SKBR3 cells and CAFs as shown in Figures 1E and 2D, respectively. Moreover, to demonstrate the specificity of BPA action, we used the environmental contaminant arsenic (Nordstrom 2002), which elicits the ability of breast cancer cells to activate ERK1/2 (Ye et al. 2005). We observed that ERK1/2 phosphorylation induced by 10 μM As_2_O_3_ was still present in SKBR3 cells transfected with shGPER (data not shown).

**Figure 3:**
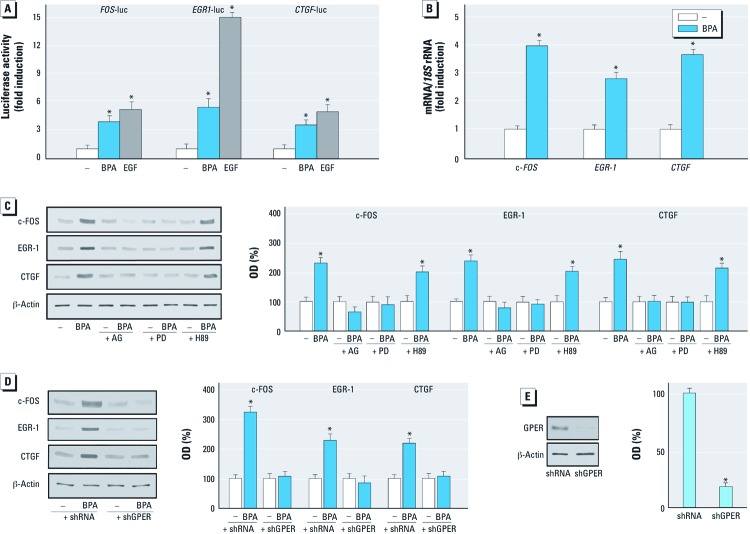
Expression of GPER target genes (*c‑FOS*, *EGR‑1*, and *CTGF*) in SKBR3 cells in response to BPA treatment. (*A*) Evaluation of *c-FOS*, *EGR‑1*, and *CTGF* luciferase reporter genes in transfected SKBR3 cells treated with vehicle (–), 1 µM BPA, or EGF (50 ng/mL; positive control). Luciferase activity was normalized to the internal transfection control; values are presented as fold change (mean ± SD) of vehicle control and represent three independent experiments, each performed in triplicate. (*B*) Evaluation of *c-FOS*, *EGR‑1*, and *CTGF* mRNA expression by real-time PCR in cells treated with 1 µM BPA for 4 hr. Gene expression was normalized to *18S* expression, and values are presented as fold change (mean ± SD) of vehicle control. (*C*) Immunoblots showing c-FOS, EGR‑1, and CTGF protein expression in SKBR3 cells treated with vehicle or 1 µM BPA alone or in combination with 10 µM AG1478, PD, or H89 (inhibitors of EGFR, MEK, and PKA respectively). (*D*) Protein levels of c-FOS, EGR-1, and CTGF in SKBR3 cells transfected with shRNA or shGPER and treated with vehicle or 1 µM BPA for 6 hr. (*E*) Efficacy of GPER silencing. Graphs show densitometric analyses of the blots normalized to β-actin; values represent the mean ± SD of three independent experiments. *p < 0.05 compared with vehicle.

**Figure 4:**
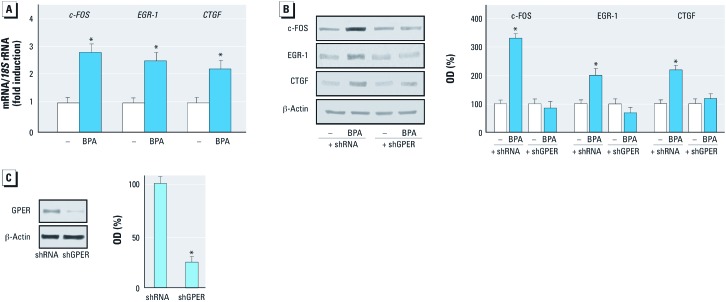
Expression of GPER target genes in CAFs in response to BPA treatment. (*A*) Evaluation of *c-FOS*, *EGR‑1*, and *CTGF* mRNA expression by real-time PCR in CAFs treated with vehicle (–) or 1 µM BPA for 4 hr. Gene expression was normalized to *18S* expression, values are presented as fold changes (mean ± SD) of vehicle control. (*B*) Expression of c-fos, EGR‑1, and CTGF protein in CAFs transfected with shRNA or shGPER and treated with vehicle or 1 µM BPA for 6 hr. (*C*) Efficacy of GPER silencing. In *B* and *C*, graphs show densitometric analyses of the blots normalized to β-actin; values represent the mean ± SD of three independent experiments. *p < 0.05 compared with vehicle.

*BPA stimulates the expression of GPER target genes.* GPER-mediated signaling regulates the transcription of diverse target genes (Pandey et al. 2009). In the present study, BPA transactivated the promoter sequence of *c-FOS*, *EGR-1*, and *CTGF* (Figure 3A), and accordingly stimulated mRNA expression of these genes (Figures 3B, 4A). In accordance with these findings, BPA induced the protein levels of c-FOS, EGR-1, and CTGF (Figure 3C). As observed with ERK1/2 activation, the EGFR inhibitor AG1478 and the ERK inhibitor PD, but not the PKA inhibitor H89, repressed the up-regulation of these proteins by BPA (Figure 3C). Notably, the c-FOS, EGR-1, and CTGF protein increases after exposure to BPA were abrogated by silencing GPER in both SKBR3 cells and CAFs (Figures 3D, 4B). The efficacy of GPER silencing was ascertained by immunoblotting experiments in SKBR3 cells and CAFs as shown in Figures 3E and 4C, respectively. Taken together, these results demonstrate that BPA regulates the expression of *c-FOS*, *EGR-1*, and *CTGF* through the GPER/EGFR/ERK transduction pathway.

**Figure 5:**
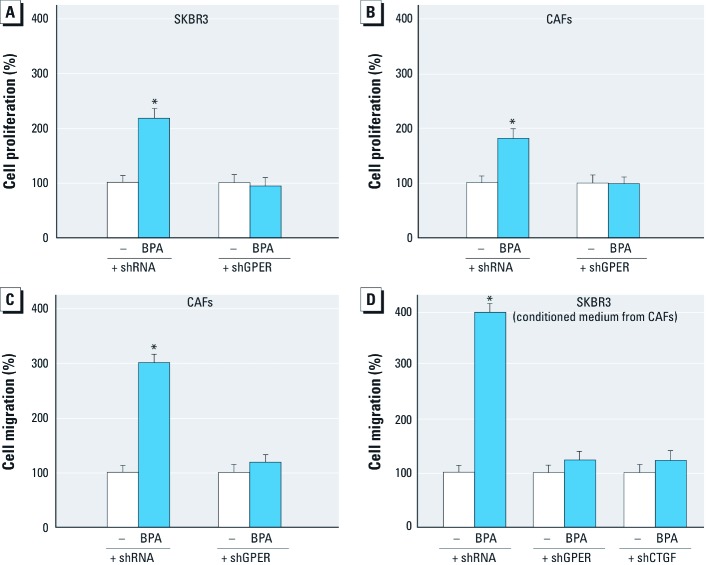
Induction of proliferation and migration in SKBR3 cells and CAFs. (*A*,*B*) Proliferation in SKBR3 cells (*A*) and CAFs (*B*) treated with vehicle (–) or 1 µM BPA for 5 days after silencing GPER expression. (*C*) Migration in CAFs treated with vehicle or 1 µM BPA for 48 hr after silencing GPER expression. (*D*) Migration in SKBR3 cells cultured in conditioned medium from CAFs with silenced expression of *GPER* and *CTGF*. Values shown represent the mean ± SD percent of vehicle control of three independent experiments, each performed in triplicate. *p < 0.05 compared with vehicle.

*BPA induces cell proliferation and migration through GPER.* The aforementioned results were recapitulated in the complex physiologic responses such as cell proliferation and migration. The proliferative effects observed in both SKBR3 cells and CAFs after 5-day treatment with BPA were cancelled when GPER expression was silenced by shGPER (Figure 5A,B). Moreover, in wound-healing assays in CAFs, migration induced by BPA was abolished by knocking down GPER expression (Figure 5C). To evaluate whether the treatment of CAFs with BPA could induce the migration of tumor cells through secreted factor(s), we performed wound-healing assays in SKBR3 cells cultured with conditioned medium from CAFs. Interestingly, the migration of SKBR3 cells was not evident after silencing GPER or CTGF expression in CAFs (Figure 5D). Overall, these findings demonstrate that BPA induces stimulatory effects as a GPER agonist in both ER-negative SKBR3 breast cancer cells and CAFs.

## Discussion

There has been increased interest in understanding the molecular mechanisms involved in the endocrine-disrupting effects of BPA (Vandenberg et al. 2009). In this regard, fetal and perinatal exposures to BPA in rodents have been shown to affect the brain, mammary gland, and reproductive tract, as well as to stimulate the development of hormone-dependent tumors (Durando et al. 2007; Munoz-de-Toro et al. 2005). Moreover, the estrogenic actions of BPA, including increased uterine wet weight, luminal epithelial height, and increased expression of the estrogen-inducible protein lactoferrin, were reported in prepubescent CD-1 mice (Markey et al. 2001). Analogously, BPA induced the proliferation of uterine and vaginal epithelial cells in ovariectomized rats (Steinmetz et al. 1998). In regard to the mechanisms by which BPA can exert estrogen-like effects, it has been reported that BPA’s two benzene rings and two (4,4´)-OH substituents fitting in the ER binding pocket allow the binding to and activation of both ERα and ERβ, which in turn mediate the transcriptional responses to BPA (Gould et al. 1998; Kuiper et al 1998; Vivacqua et al 2003). In addition, rapid nongenomic effects involving diverse transduction pathways were observed upon exposure to BPA in pancreatic islet, endothelial, and hypophysial cells and in breast cancer cells (Alonso-Magdalena et al. 2005; Noguchi et al. 2002; Watson et al. 2007). In this context, the novel estrogen receptor GPER was recently shown to mediate the BPA-dependent rapid activation of intracellular signaling (Dong et al. 2011) and the proliferation of both human seminoma cells (Bouskine et al. 2009) and mouse spermatogonial cells (Sheng and Zhu 2011).

To investigate the potential of GPER to mediate estrogenic signals such as those elicited by BPA, we used SKBR3 breast cancer cells and CAFs, both of which express GPER and lack ERs. Interestingly, we found that in both cell types BPA triggers rapid ERK activation through the GPER/EGFR transduction pathway and induces the expression of genes that characterize estrogenic GPER-mediated signaling (Pandey et al. 2009). In addition, we determined that BPA stimulates the proliferation and migration of SKBR3 cells and CAFs through GPER. Of note, conditioned medium from BPA-treated CAFs induced the migration of SKBR3 cells, suggesting that BPA may also promote a functional crosstalk between cancer cells and CAFs. These data regarding CAFs are particularly intriguing given that these cells actively contribute to cancer growth and progression even at metastatic sites (Bhowmick and Moses 2005).

The present findings are relevant to the results obtained in a previous study (Albanito et al. 2008) in which we found that atrazine, another environmental contaminant, triggered estrogen-like activity through the GPER/EGFR/ERK transduction pathway in hormone-sensitive ovarian cancer cells. Moreover, in that study (Albanito et al. 2008) we observed that atrazine induced functional crosstalk between GPER and ERα in accordance with the results of Sheng and Zhu (2011) who demonstrated a similar interaction in mouse spermatogonial cells after exposure to BPA. Overall, these findings, together with results of the present study, contribute to a better understanding on the multifaceted mechanisms by which environmental estrogens may act as endocrine stimulators in hormone-dependent malignancies.

BPA is consistently detected in almost all individuals in developed nations (Welshons et al. 2006), suggesting that humans are exposed to BPA continuously. In addition, the rapid metabolic clearance of BPA and its detectable levels in human blood and urine suggest that the intake of BPA may be higher than indicated by diverse studies and that long-term daily intake may lead to its bioaccumulation. In this regard, previous studies (Vandenberg et al. 2009) have estimated that human exposure ranges from < 1 μg/kg/day to almost 5 μg/kg/day (0.325 mg/day/adult). However, pharmacokinetic modeling data have shown that oral intakes up to 100 mg/day/adult would be required to explain the reported human circulating levels (Vandenberg et al. 2009). Hence, future studies should include mathematical models of potential exposures, particularly because many sources of BPA exposure have been identified (Vandenberg et al. 2009). These observations suggest that the BPA concentration used in the present study is achievable in humans. In the present study, we found that BPA is able to trigger GPER-mediated signaling in breast cancer cells and CAFs, which contributes to tumor progression. Thus, GPER may a potential mediator of the estrogen-like activity of BPA, as well as a further biological target in estrogen-sensitive tumors.

## Supplemental Material

(172 KB) PDF
