# Supplementary material for: Bisphenol A Induces Gene Expression Changes and Proliferative Effects through GPER in Breast Cancer Cells and Cancer-Associated Fibroblasts
Source: Environ Health Perspect. 2012 May 2;120(8):1177–82. doi: 10.1289/ehp.1104526 (PMC3440081; doi:10.1289/ehp.1104526)
Supplement: (172 KB) PDF [file ehp.1104526.s001.pdf]

Supplemental Material

Bisphenol A Induces Gene Expression Changes and Proliferative Effects through GPER in Breast Cancer Cells and Cancer-Associated Fibroblasts.

Marco Pupo[1], Assunta Pisano[1], Rosamaria Lappano[1], Maria Francesca Santolla[1], Ernestina Marianna De Francesco[1], Sergio Abonante[2], Camillo Rosano[3], Marcello Maggiolini[1]

[1]Department of Pharmaco-Biology University of Calabria, Rende, Italy. [2]Regional Hospital, Cosenza, Italy. [3]Department of Bioinformatics and Structural proteomics, National Institute for Cancer Research, Genova, Italy.

Table of Contents

Supplemental Material, Table 1 ....PAGE 3

Supplemental Material, Figure 1 ....PAGE 4

Supplemental Material, Table S1: Sequences of primers used

Gene	Primer Fw	Primer Rv
c-FOS	5'-CGAGCCCTTTGATGACTTCCT-3'	5'-GGAGCGGGCTGTCTCAGA-3'
CTGF	5'-ACCTGTGGGATGGGCATCT-3'	5'-CAGGCGGCTCTGCTTCTCTA-3'
EGR-1	5'-GCCTGCGACATCTGTGGAA-3'	5'-.CGCAAGTGGATCTTGGTATGC-3'
18S	5'-GCGTCCCCCAACTTCTTA-3'	5'-GGGCATCACAGACCTGTTATT-3'

Supplemental Material, Figure S1

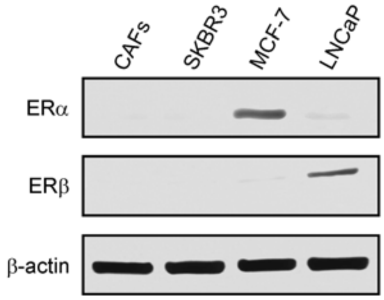

CAFs and SKBR3 cells are ERα and ERβ negative. Western blot analyses of ERα and ERβ protein expression in CAFs, SKBR3 and MCF-7 breast cancer cells and LNCaP prostate cancer cells. β-actin antibody was used as a loading control.
